# Characteristics of prostate biopsy in patients under the dutasteride treatment

**DOI:** 10.1097/MD.0000000000031658

**Published:** 2022-11-04

**Authors:** Daisuke Obinata, Ken Nakahara, Tsuyoshi Yoshizawa, Junichi Mochida, Kenya Yamaguchi, Satoru Takahashi

**Affiliations:** a Department of Urology, Nihon University School of Medicine, Tokyo, Japan.

**Keywords:** cancer detection rate, dutasteride, MRI, prostate cancer, PSA

## Abstract

We performed a retrospective study to clarify the characteristics of prostate biopsies in patients treated with dutasteride, a benign prostate hyperplasia treatment drug that inhibits 5α-reductase. We studied the digital clinical data of 677 patients, including 96 cases treated with dutasteride, with suspected localized prostate cancer. All patients underwent transrectal ultrasonography-guided prostate biopsy between 2014 and 2017 in our department. A propensity score matching analysis was performed based on prostate-specific antigen (PSA) (calculated as double the PSA value for the dutasteride group) and age. Ninety-six patients in each of the dutasteride and control groups were assessed and their characteristics were compared. The characteristics of the patients in the dutasteride and control groups were well balanced by matching. There were fewer prostate cancer-positive patients in the dutasteride group. When comparing only the prostate cancer-positive patients in each group, there were significantly more cases of high-grade cancers and abnormal magnetic resonance imaging (MRI) findings in the dutasteride group. In the dutasteride group, abnormal MRI findings and advanced age were significant predictors of high grade cancer. This study shows the characteristics of prostate biopsies in patients treated with dutasteride and indicates that patients on dutasteride with advanced age and abnormal MRI findings should undergo prostate biopsy.

## 1. Introduction

The Japanese Association of Cancer Registries showed that the incidence of localized prostate cancer (PCa) in Japan increased to the 4th most common cancer in 2018.^[1]^ Although advanced PCa has become a serious disease, early detection and characterization of PCa allows for more treatment options and has led to improved specific survival. A combination of measurement of prostate-specific antigen (PSA) concentrations in blood with a prostate biopsy is known to be the standard diagnostic technique for PCa.^[2]^ Standard biopsy is performed systemically, taking at least 8 cores according to the sextant prostate biopsy method.^[3]^ The detection rate by biopsy is low as 25% of patients with PSA lab results show PSA values between 2 and 10 μg/L.^[4,5]^ Although there is still no consensus on the adequate core number required for biopsy, it is well known that increasing the core number elevates the cancer detection rate.^[3]^

The benign prostate hyperplasia (BPH) treatment drug dutasteride, a 5α-reductase (5AR) inhibitor, is well known to reduce prostate carcinogenesis and PSA.^[6]^ 5AR inhibitors block the conversion of testosterone to 5‐α‐dihydrotestosterone, which induces prostate epithelial cell proliferation. There are 2 types of 5Ars. The presence of type 1 promotes Pca carcinogenesis, whereas type 2 does not.^[7]^ Dutasteride is known to block both type 1 and 2, while another 5AR inhibitor finasteride blocks type 2 only.^[8]^ Despite this, 5AR inhibitors have been reported to increase clinically significant cancer rates. Thus, the use of dutasteride for Pca chemoprevention remains controversial.^[9]^ In addition, since dutasteride reduces PSA levels by an average of about 50%, it is recommended that PSA levels in dutasteride-treated patients be evaluated at twice the PSA level,^[10]^ making prostate biopsy decisions based on PSA levels more difficult. Previously, we reported that high PSA density, low prostate volume reduction, and positive abnormal magnetic resonance imaging (MRI) findings are significant predictive factors for Pca in BPH patients treated with dutasteride.^[11]^ However, our previous study was based on a small number of patients and did not evaluate comparative studies with patients not treated with dutasteride.^[11]^ Also, the evaluation of biopsy core positivity rates was unknown in our previous study.^[11]^ In this study, we have retrospectively compared the characteristics of prostate biopsies in BPH patients treated with dutasteride and in untreated patients, adjusted for PSA levels and age.

## 2. Material and methods

We reviewed the digital clinical data of 677 Asian PCa suspects who underwent transrectal ultrasonography-guided prostate biopsies in our department from 2014 to 2017. Ninety-six of these patients were treated with dutasteride for BPH. Before the biopsy, 553 patients underwent diagnostic prostate multi-parametric MRI (mpMRI) on a 3-Tesla or 1.5-Tesla MR scanner using T2-weighted imaging and diffusion-weighted imaging. The radiologists in our hospital evaluated mpMRI images based on the prostate imaging and reporting data system scoring system versions I and II. All patients underwent a standard systematic biopsy. High grade PCa was defined as a Gleason score of 8 or higher. Patient characteristics were compared between patients with or without dutasteride treatment (dutasteride vs control group). A propensity score matching (PSM) analysis was performed based on PSA levels (calculated as double for the dutasteride group) and age. Ninety-six patients in each of the dutasteride and control groups were evaluated, and their characteristics were compared.

Analyses were performed using SPSS statistics (IBM Japan, Tokyo, Japan) and JMP® version 9 (SAS Institute Japan, Inc., Tokyo, Japan). Continuous data are presented as mean ± standard of error. Student *t*-test with Cohen’s *d* was used for the differences in continuous data across dichotomous categories. The validity of the sample size required for the t-test was evaluated using G*power.^[12]^ Setting the effect size to 0.5, alpha error to 0.05, power to 0.8, and 2-sided test, the required sample size was 64. Chi-square and Fisher exact tests were used for categorical variables. Univariate and multivariate logistic regression analyses were used to identify the appropriate factors for high grade PCa detection. The area under the receiver operating characteristic curve was evaluated for high grade PCa detection. Statistical significance was set at *P* < .05. Cancer specific survival analyses were conducted according to the Kaplan–Meier method and survival characteristics were compared using the log-rank test. This study was approved by the Institutional Review Board and Research Ethics Committee of Nihon University School of Medicine (RK-190611-3) and all study participants provided informed consent.

## 3. Results

Patient backgrounds of both groups before and after PSM are shown in Table 1 (Table S1, Supplemental Digital Content, http://links.lww.com/MD/H866). Almost all patients (93 of 96 in the dutasteride and 573 of 581 in the control group) underwent prostate biopsy for the 1st time. Before PSM, patients tended to be in a higher age group and have a lower PSA compared to controls (73.40 vs 70.56, *P* = .002, 12.76 vs 135.31, *P* < .001, Table S1, Supplemental Digital Content, http://links.lww.com/MD/H866). After PSM, age and PSA (calculated as double in the dutasteride group) included in the PS model were well balanced (Table 1). As before PSM, there were no significant differences between groups in the proportion of the cases with MRI-performed before the biopsy and prostate cancer-detection (Table 1).

**Table 1:** Asian patient characteristics after propensity score matching.

	Dutasteride (n = 96)	Control (n = 96)	*P* value	Cohen *d*
Repeated biopsy cases	3	0	.08	-
Age (SD) years	73.40 (7.92)	72.48 (8.28)	.43	0.11
PSA (SD) ng/ml	12.76 (15.64)	19.71 (44.32)	.15	0.20
MRI	77	79	.71	-
Prostate cancer	52	64	.15	-
Gleason score			.22	-
6	5	13		
7	15	24		
8	16	14		
9	15	14		
10	1	0		

MRI = magnetic resonance imaging, PSA = prostate-specific antigen, SD = standard deviation.

**Figure 1.**
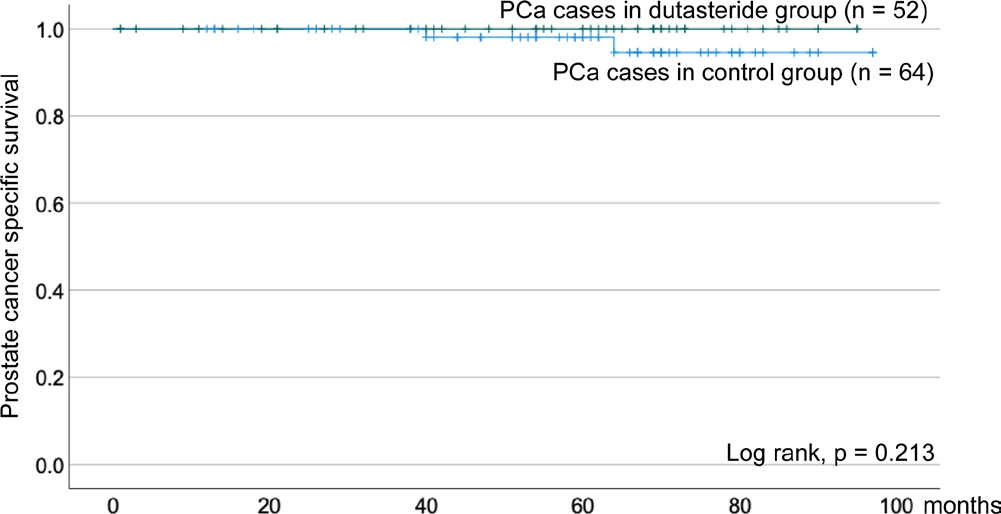
Kaplan–Meier estimates of cancer specific survival with/ without dutasteride treatment.

From the matched cohort, PCa-positive cases were selected and compared between the 2 groups (52 and 64 cases). Dutasteride group had significantly higher rates of abnormal MRI findings and high grade Pca cases (39 of 52 vs 37 of 64, *P* = .024, 32 of 52, 27 of 64, *P* = .038, respectively, Table 2). However, there were no significant differences in the biopsy-positive core rate or clinical N and M stages (0.30 vs 0.33, *P* = .58, N1 and M1 cases: 3 and 1 of 52 vs 5 and 7 of 64, *P* = .66 and *P* = .057, respectively). Furthermore, cancer-specific survival rates were compared, but no significant differences were found (Fig. 1, *P* = .213).

**Table 2:** Asian patient characteristics in prostate cancer cases after propensity score matching.

	Dutasteride (n = 52)	Control (n = 64)	*P* value	Cohen *d*
MRI abnormal finding cases	39	37	.024	-
Gleason score 8 or more	32	27	.038	-
Cancer positive core rate (SD)	0.30 (0.22)	0.33 (0.26)	.58	0.1
Clinical N1 stage	3	5	.66	-
Clinical M1 stage	1	7	.057	-

MRI = magnetic resonance imaging, SD = standard deviation.

Next, we performed univariate and multivariate logistic regression analyses of factors associated with prostate cancer with a high Gleason score in dutasteride group. Age and abnormal MRI findings, but not PSA, were statistically independent predictors of high grade PCa (odd ratio = 1.089, *P* = .022, and odds ratio = 5.681, *P* = .007, respectively, Table 3). The area under the curve (AUC) of each parameter was age, 0.71 (95% confidence interval [CI]: 0.60 – -0.82, *P* = .001) and abnormal MRI findings, 0.69 (95% CI: 0.57 – 0.81, *P* = .005). Based on this AUC, the optimum cutoff value from the Youden index, sensitivity, specificity, and positive and negative predictive values (PPV and NPV) at an age were evaluated. The optimum cutoff value of age was 72.5, with a sensitivity of 0.68, specificity of 0.52, PPV of 41%, and NPV of 76.7%.

**Table 3:** Univariate logistic regression analysis of factors associated High Gleason score prostate cancer in Asian dutasteride cases (n = 96).

	Univariate			Multivariate		
Variable	Odds ratio	95% index	*P* value	Odds ratio	95% index	*P* value
Age	1.07	1.040–1.187	.002	1.089	1.012–1.171	.022
PSA	1.05	1.009–1.096	.016	1.026	0.977–1.077	.306
Abnormal MRI findings	6.75	2.036–22.38	.002	5.681	1.614–19.996	.007

MRI = magnetic resonance imaging, PSA = prostate-specific antigen.

## 4. Discussion

In this study, we investigated the characteristics of prostate biopsies in patients with BPH treated with dutasteride. Andriole et al reported that dutasteride reduced the risk of incident PCa detected on biopsy; however, routine prostate biopsies detected PCa in approximately 19% of the dutasteride patients and 25% of the placebo patients,^[10]^ which is lower than the positive rate in the present study. This might be because the indications for biopsy in this study were limited to patients where PCa was more strongly suspected. Interestingly, consistently with a previous report,^[10]^ high grade PCa cases were significantly more common in the dutasteride group compared to the control group matched with doubled PSA. Recently, Yoo et al reported the ability to predict the probability of pathologic upgrading or downgrading based on the proportion of core biopsies with the highest Gleason grade.^[13]^ They suggested that the percentage of positive cores with the highest Gleason score was associated with PCa progression. In our data, the mean positive core rate was similar in both groups, at 30%. Although the high grade Gleason score is a significant prognostic factor for prostate cancer,^[14]^ there were no significant differences the in clinical stages or cancer-specific survival in this study. These data suggest that dutasteride could have inhibitory effects on prostate cancer and that appropriate treatment with early detection has a favorable prognostic impact. Therefore, it is important to identify significant predictors to determine the indication for a biopsy in dutasteride-treated patients.

In addition to previous studies that reported that PSA density, prostate volume reduction rate, and MRI findings are important predictive factors for PCa detection,^[11]^ we found that advanced age and abnormal MRI findings were clinically significant predictors of high grade PCa in BPH patients treated with dutasteride. The relationship between prostate cancer grade and age is controversial^[15]^; however, in this study, age was found to be associated with high-grade prostate cancer in patients treated with dutasteride. mpMRI of the prostate is important for the diagnosis and staging of PCa. Following the development of the prostate imaging and reporting data system version 1 and 2 by the European Society of Urogenital Radiology,^[16,17]^ mpMRI allows the clinician to determine prostate biopsy for patients with abnormal PSA and target the clinically important region within the prostate.^[18,19]^ Interestingly, PSA and advanced age are known to be closely related to high grade PCa,^[20]^ however, in the present study, PSA was not a significant factor, which may be a feature of BPH treated with dutasteride.

This study has several limitations, including a retrospective design, small patient sample, and lack of racial diversity (only Japanese cases were evaluated). The sample size was calculated from the National Database of Health Insurance Claims and Specific Health Checkups of Japan (NDB open data: (https://www.mhlw.go.jp/stf/seisakunitsuite/bunya/0000177182.html) of the Japanese population consuming dutasteride, which was approximately 3,30,000 persons. The sample size of 96 corresponded to a CI of 95% and an acceptable margin of error of 10%. Similarly, the total number of prostate biopsies in Japan during the 4-years observation period of this study, calculated from the National Database of Health Insurance Claims and Specific Health Checkups of Japan, has been estimated to be about 4,00,000, and the 581 prostate biopsies in this study correspond to a CI of 95% and a margin of error of 4%. The margin of error of the dutasteride group was slightly larger at 10%, which is a limitation of this study.

## 5. Conclusions

Dutasteride has been shown to have a variety of effects on prostate cancer; however, MRI findings were found to be similar to those in the treatment-naïve patients. On the other hand, as previously reported,^[10]^ higher-grade cancers were more common in the dutasteride-treated patients, but there was no difference in cancer-specific survival rates, suggesting the importance of early detection. This study suggests that prostate biopsies should be considered in elderly dutasteride-treated patients with abnormal MRI findings regardless of PSA levels. Further studies are required to verify these findings.

## Acknowledgments

The authors thank Ms. Emiko Kuriki, MR. Mitsuo Kano, and MS. Yuki Ikemori for assistance of this study. The authors would also like to thank Dr Seiichi Udagawa for his statistical advice.

## Author contributions

**Conceptualization:** Daisuke Obinata, Kenya Yamaguchi, Satoru Takahashi.

**Data curation:** Ken Nakahara, Tsuyoshi Yoshizawa, Junichi Mochida.

**Funding acquisition:** Daisuke Obinata.

**Investigation:** Kenya Yamaguchi.

**Methodology:** Daisuke Obinata.

**Project administration:** Daisuke Obinata, Kenya Yamaguchi.

**Supervision:** Satoru Takahashi.

**Writing – original draft:** Daisuke Obinata.

**Writing – review & editing:** Kenya Yamaguchi, Satoru Takahashi.

## Supplementary Material


