# Supplementary material for: Characteristics of prostate biopsy in patients under the dutasteride treatment
Source: Medicine (Baltimore). 2022 Nov 4;101(44):e31658. doi: 10.1097/MD.0000000000031658 (PMC9646501; doi:10.1097/MD.0000000000031658)
Supplement: Supplementary file 1 [file medi-101-e31658-s001.pdf]

Supplementary table. Asian patient characteristics before propensity score matching (n = 677).

	Dutasteride (n=96)	Control (n=581)	P value	Cohen's *d*
Repeated biopsy cases	3	8	0.20	-
Age (SD) years	73.40 (7.92)	70.56 (9.38)	0.002	0.30
PSA (SD) ng/ml	12.76 (15.64)	135.31 (712.28)	< 0.001	0.18
MRI	77	476	0.68	-
Prostate cancer	52	364	0.11	-
Gleason score			0.52	-
6	5	59		
7	15	108		
8	16	86		
9	15	94		
10	1	17		

MRI: magnetic resonance imaging

PSA: prostate-specific antigen

SD: standard deviation
